# Microwave-Assisted Extraction of Phlorotannins from *Fucus vesiculosus*

**DOI:** 10.3390/md18110559

**Published:** 2020-11-15

**Authors:** Sónia J. Amarante, Marcelo D. Catarino, Catarina Marçal, Artur M. S. Silva, Rita Ferreira, Susana M. Cardoso

**Affiliations:** LAQV-REQUIMTE, Department of Chemistry, University of Aveiro, 3810-193 Aveiro, Portugal; sonia.amarante@ua.pt (S.J.A.); mcatarino@ua.pt (M.D.C.); catarina.marcal@ua.pt (C.M.); artur.silva@ua.pt (A.M.S.S.); ritaferreira@ua.pt (R.F.)

**Keywords:** brown seaweeds, phlorotannins, microwave-assisted extraction, response surface methodology, antioxidant, antiradical activity, xanthine oxidase, α-glucosidase

## Abstract

Microwave-assisted extraction (MAE) was carried out to maximize the extraction of phlorotannins from *Fucus vesiculosus* using a hydroethanolic mixture as a solvent, as an alternative to the conventional method with a hydroacetonic mixture. Optimal MAE conditions were set as ethanol concentration of 57% (*v/v*), temperature of 75 °C, and time of 5 min, which allowed a similar recovery of phlorotannins from the macroalgae compared to the conventional extraction. While the phlorotannins richness of the conventional extract was slightly superior to that of MAE (11.1 ± 1.3 vs. 9.8 ± 1.8 mg PGE/g DW_extract_), both extracts presented identical phlorotannins constituents, which included, among others, tetrafucol, pentafucol, hexafucol, and heptafucol structures. In addition, MAE showed a moderate capacity to scavenge ABTS^•+^ (IC_50_ of 96.0 ± 3.4 µg/mL) and to inhibit the activity of xanthine oxidase (IC_50_ of 23.1 ± 3.4 µg/mL) and a superior ability to control the activity of the key metabolic enzyme α-glucosidase compared to the pharmaceutical drug acarbose.

## 1. Introduction

Seaweeds are claimed to be a sustainable and rich source of bioactive compounds, holding huge application potential in distinct fields. Among seaweeds’ bioactive compounds, phlorotannins—i.e., phenolic compounds typical from brown macroalgae—are one of the most promising, since they have been related to numerous beneficial biological properties, including antioxidant [1,2,3], anti-inflammatory [4,5], antibacterial [6], anticancer [7], and antidiabetic [8] activities. While not fully elucidated, the bioactivity of phlorotannins is accepted as being largely dependent on their structure.

Chemically, these compounds consist of dehydro-oligomers or dehydro-polymers formed through the C-C and/or C-O-C oxidative coupling of phloroglucinol (1,3,5-trihydroxybenzene), which may occur in a wide range of molecular sizes and in different assemblages [9,10]. According to the number of hydroxyl groups and the nature of the structural linkages between phloroglucinol units, they are classified in four groups: phlorethols and fuhalols (containing ether linkages), fucols (containing aryl linkages), fucophlorethols (containing both aryl-aryl and ether linkages), and eckols and carmalols (containing dibenzodoxine linkages) [11].

*Fucus vesiculosus* is a widespread species, which is naturally found along the coastlines of the North Sea, the western Baltic Sea, and the Atlantic and Pacific oceans [12]. Phlorotannins-rich extracts obtained from *F. vesiculosus* have been described for their promising antioxidant, anti-inflammatory, and antitumor activities among other things, granting them great potential for application in the food, cosmetic, and pharmaceutical industries [12].

As for tannins in general, the extraction of phlorotannins is traditionally performed by the conventional solvent extraction method [8,12,13], using hydroacetonic mixtures, although some authors have also resorted to hydroethanol and hydromethanol mixtures [14,15,16]. Due to their peculiar characteristics including chemical complexity, susceptibility to oxidation, and interaction with other components of the matrix, the extraction of phlorotannins is a challenging process and the structures found in crude extracts and in purified fractions may depend on the extraction conditions applied [11,17,18].

In addition to the traditional solid–liquid extraction at room temperature, advanced methods such as supercritical fluid extraction (SFE) [19,20], pressurized liquid extraction (PLE) [21], microwave-assisted extraction (MAE) [12,22], and ultrasound-assisted extraction (UAE) [23,24] have been previously used for recovery of phlorotannins from seaweeds. Nowadays, MAE is one of the techniques that allow fast and large extraction of bioactive compounds, including phenolic compounds [12], showing several advantages over other methods. Among others, it allows the rapid heating of aqueous samples with non-ionizing electromagnetic radiation, a lower solvent use, a greater selectivity for the family of compounds of interest, a higher level of automation, a superior efficiency, and lower extraction times [22]. Since several variables influence the extraction of phlorotannins, the optimal operating extraction parameters may be estimated with a statistical optimization method. The response surface methodology (RSM) makes use of the quantitative data of an appropriate experimental design to determine and simultaneously solve the multivariate equation. In order to minimize the number of experiments, this methodology relies on a mathematical model where all the interactions that occur between the test variables are taken into account [25]. This type of approach enables a considerable reduction in the cost and execution time in experimental projects with more than two variables [26]. One of the RSM models most used for experimental planning is the Box–Behnken design (BBD). The main advantage of this experimental design is that the experiments are not carried out under extreme conditions—i.e., the combinations between the different factors are never in their higher or lower levels, since this type of combination usually gives unsatisfactory results [27]. As far as we know, previous studies focusing on the extraction of phlorotannins by MAE have already been applied in seaweeds from the *Saccharina*, *Carpophyllum*, and *Ecklonia* genera, but no study has been performed with *Fucus* genus yet.

In this context, this study aimed to optimize the extraction process of phlorotannins from *F. vesiculosus* using the MAE technique and a green solvent—namely, ethanol. In addition, it was intended to elucidate the potential biological capacity of the resultant extracts, particularly with respect to their ability to act against oxidative events and to control the activity of α-glucosidase (i.e., a key enzyme in diabetes control). All the data were compared with those obtained by the conventional method using hydroacetonic mixtures.

## 2. Results

### 2.1. Single-Factor Experiment on MAE

**Figure 1:**
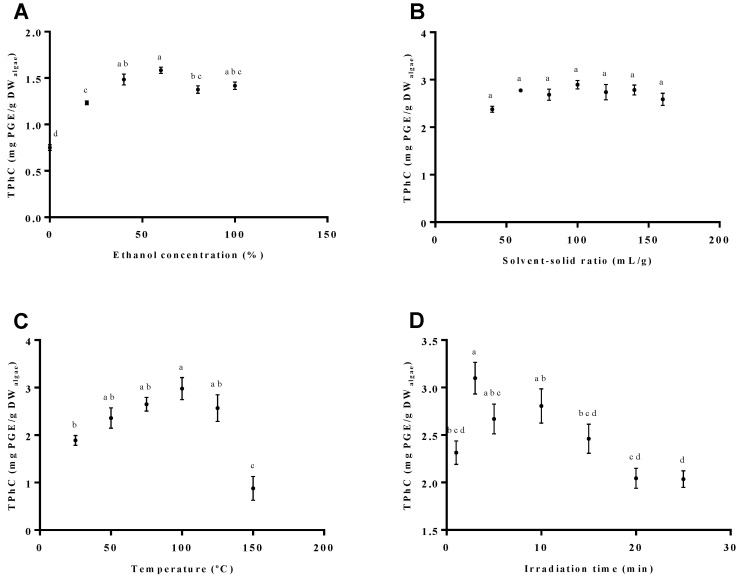
Effect of (**A**) ethanol concentration, (**B**) solvent–solid ratio, (**C**) temperature, and (**D**) irradiation time on the recovery of phlorotannins from *F. vesiculosus* in the single-factor experiments. Data represent the mean ± SEM and the results are expressed in mg of phloroglucinol equivalents/g of dried algae (mg PGE/g DW_algae_). Different letters represent statistical significance (one-way ANOVA followed by Tukey’s post hoc test; *p* ≤ 0.05).

Taking into account the different variables that could mainly affect the phlorotannins extraction, preliminary single-factor experiments were performed to specify the selected factors in the BBD experiment. Different concentrations of ethanol were tested in the range of 0% to 100% (*v/v*). According to Figure 1A, the total phlorotannins content (TPhC) recovered from *F. vesiculosus* increased almost proportionally between 20% and 60% ethanol (1.23 ± 0.03 to 1.59 ± 0.03 mg PGE/g DW_algae_), with the maximum yield obtained for this last concentration. In turn, the use of ethanol above 60% resulted in a decrease in the TPhC to approximately 1.40 mg PGE/g DW_algae_. Based on this, the concentration of ethanol used to study the next variable was 60%. Moreover, considering these results, an ethanol concentration range between 40% and 80% was selected for the BBD experiment.

The effect of different solvent–solid ratios on the TPhC recovered from *F. vesiculosus* was tested in the range of 40 to 160 mL/g, as for our previous study [8]. As represented in Figure 1B, the variation in this parameter did not significantly influence the TPhC, which accounted for approximately 2.7 mg PGE/g of DW_algae_ from 60 to 160 mL/g. Yet, given that a maximum point was achieved at 100 mL/g (2.90 ± 0.09 mg PGE/g DW_algae_), this solvent–solid ratio was selected for the following factor study and for the BBD experiment as well.

It is expected that temperature affects the extraction process of thermolabile compounds such as phlorotannins. Taking this into account, different temperatures were selected between 25 and 150 °C. As represented in Figure 1C, a linear increase in the recovery of TPhC was obtained between 25 and 100 °C (1.89 ± 0.10 to 2.98 ± 0.24 mg PGE/g DW_algae_). However, temperatures of extraction above 100 °C—namely, 125 and 150 °C—caused a decrement of the phlorotannins recovery (TPhC of 2.57 ± 0.28 and 0.878 ± 0.251 mg PGE/g DW_algae_, respectively). Hence, for the analysis of the next variable, the temperature was set at 100 °C, which presented the maximum TPhC yield. Additionally, for the BBD experiment, the interval chosen was 75–125 °C.

Moreover, based on the literature data for other algae [12,22], the influence of the irradiation time was considered for the interval of 1 to 25 min. As depicted in Figure 1D, while the raising of the irradiation time up to 3 min caused an increase in the amount of TPhC (maximum 3.10 ± 0.17 mg PGE/g DW_algae_), the opposite tendency was registered for longer extraction periods, reaching levels of 2 mg PGE/g of DW_algae_ for 20 min of extraction time. Based on these results, the irradiation time interval selected for the BBD experiment was 1–5 min.

### 2.2. Analysis of the Response Surface Methodology

#### 2.2.1. Fitting the Model

The experimental values obtained for TPhC, represented in Table 1, were fitted to a quadratic polynomial model (Equation (1)). This equation allowed the determination of the optimal conditions for the extraction process in order to obtain the maximum phlorotannins recovery and also to determine the different correlations, which are related to the independent variable interactions and respective responses.

The experimental data allowed the determination of the coefficients of the model, which were evaluated for statistical significance using a statistical analysis of variance (ANOVA) and are listed in Table 2. Accordingly, the independent variables with a higher impact on TPhC were the temperature (*X*_2_, *p* < 0.001) and time (*X*_3_, *p* < 0.01), while the ethanol concentration revealed no effect. Moreover, significant interactive effects between the ethanol concentration and temperature (*X*_1_*X*_2_, *p* < 0.05) and between the temperature and time (*X*_2_*X*_3_, *p* < 0.001) were observed. The variables ethanol concentration and temperature also showed a significant quadratic effect on TPhC (*p* < 0.01, for both).

(1) $$TPhC=2.58-0.004X_{1}-0.52X_{2}-0.24X_{3}-0.36X_{1}{}^{2}-0.38X_{2}{}^{2}-0.0001X_{3}{}^{2}+0.17X_{1}X_{2}+0.073X_{1}X_{3}-0.36X_{2}X_{3}.$$

The statistical analysis revealed a high *F*-value (43.77) and, simultaneously, a low *p*-value (*p* < 0.001), meaning that the model is significant. Furthermore, the coefficient of multiple determination (*R*^2^) for the response TPhC was 0.99 and the adjusted determination coefficient (*R*^2^Adj) was 0.96. The similarity of these values suggests that there is a good correlation between the observed and predicted values for TPhC. Taking this into account, the fitted model may be assumed as trustworthy and capable of predicting the TPhC response.

#### 2.2.2. Effect of the Independent Variables on TPhC

**Figure 2:**
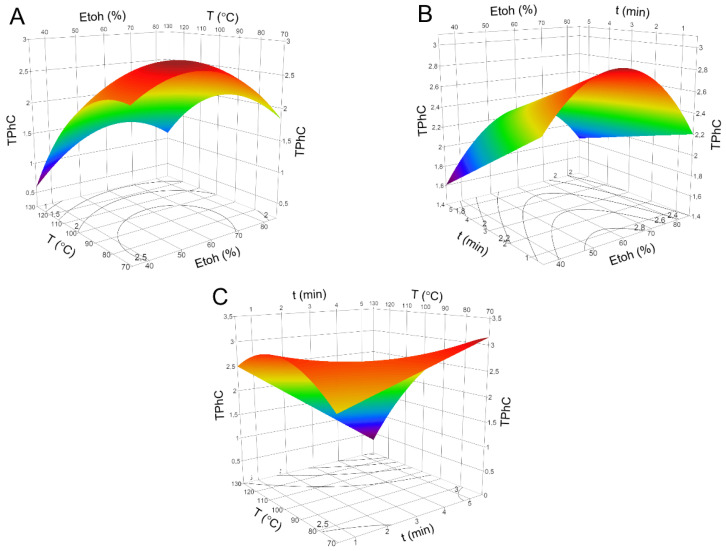
Response surface and contour plots for the total phlorotannins content (TPhC, expressed as mg of phloroglucinol equivalents/g of dried algae ie, mg PGE/g DW_algae_) from *F. vesiculosus* extracts with respect to (**A**) ethanol concentration (%, *X*_1_) and temperature (°C, *X*_2_); (**B**) ethanol concentration (%, *X*_1_) and time (min, *X*_3_); and (**C**) temperature (°C, *X*_2_) and time (min, *X*_3_). The third variable of each graph was kept at its zero level.

The effects of the independent variables and their mutual interactions on TPhC can be visualized on the three-dimensional response surface plots and two-dimensional contour plots shown in Figure 2, respectively. Each plot demonstrates the effects of two independent variables on the target response, while the third variable is maintained at its zero level.

According to the results of the regression coefficient shown in Table 2, the interaction between the ethanol concentration and temperature, and temperature and time, revealed a significant effect on the TPhC (*p* < 0.05 and *p* < 0.001, respectively). As observed in Figure 2A, this increased in the range of temperatures between 125 and 75 °C and of ethanol between 50% and 60%. In addition, both independent variables, ethanol concentration and temperature, had a significant quadratic effect (*p* < 0.01, for both). Figure 2B also demonstrated an increase in TPhC for short time extractions (1 to 5 min) and a quadratic effect for ethanol concentrations between 50% and 70%. Moreover, a higher level of TPhC was obtained at lower temperatures (75 °C) and for a longer time interval (5 min) (Figure 2C). In addition, the quadratic effect of temperature was also registered in this figure.

#### 2.2.3. Optimization and Validation of the Models

The optimal MAE conditions for the extraction of phlorotannins from *F. vesiculosus* were estimated according the quadratic polynomial model (Equation (1)). The settled conditions were ethanol concentration at 57% (*v/v*), temperature at 75 °C, and time at 5 min, with a theoretical maximum value of TPhC of 3.01 ± 0.25 mg PGE/g DW_algae_. These conditions were tested to validate the adequacy of the model prediction, and the experimental value of 3.16 ± 0.06 mg PGE/g DW_algae_ was obtained, thus demonstrating a good correlation between the experimental and predicted values, confirming the appropriateness of this model, which is trustworthy and precise.

### 2.3. Comparison between MAE and Conventional Solvent Extraction

To conclude on the feasibility of using MAE and ethanol as an alternative extraction method for the extraction of phlorotannins from *F. vesiculosus*, the TPhC (mg PGE/g DW_algae_) was compared to that of an extract obtained by conventional extraction under optimized conditions, as previously established by our group for this macroalgae species [8]—i.e., a solvent–solid ratio of 70 mL/g with 70% acetone and 1% glacial acetic acid at room temperature for 3 h. Moreover, the extracts’ richness in phlorotannins and their potential to hamper oxidative events and the activity of α-glucosidase (a key metabolic enzyme in the control of diabetes) were compared.

#### 2.3.1. Phlorotannins

Using optimal conditions, it was possible to recover similar amounts of phlorotannins with the two methods (3.16 ± 0.06 mg PGE/g DW_algae_ versus 2.94 ± 0.28 mg PGE/g DW_algae_ for MAE and conventional extraction, respectively). On the other hand, when comparing the phlorotannins richness of both extracts, it is possible to conclude that the MAE had a slightly lower concentration of phlorotannins (Table 3), suggesting that, compared to the conventional extraction using hydroacetone, the application of MAE at 75°C and ethanol concentration of 57% can further facilitate the co-extraction of non-phenolic components—most likely, lipophilic compounds such as fatty acids, sterols, and pigments (in particular, fucoxanthin—i.e., the characteristic pigment from brown algae). Nevertheless, the individual phlorotannins detected by UHPLC-DAD-ESI-MS^n^ were, in general, coincident between the two extracts (Table 3).

#### 2.3.2. Bioactive Potential

The bioactive capacity of the two extracts was evaluated for their capacity to act as antioxidant and antidiabetic agents—namely, through the ability to scavenge radicals (ABTS^•+^ and O_2_^•−^), inhibit the activity of xanthine oxidase, and control the activity of α-glucosidase. Consistent with the phlorotannins’ superior richness in conventional extract compared to MAE, in general the latter was less active (Table 4). Despite this, it is worth mentioning that this extract had a promising potential to hamper the activity of xanthine oxidase and, in particular, of α-glucosidase, for which the IC_50_ was 115 times lower than that of the commercial drug acarbose.

## 3. Discussion

Since MAE has several advantages compared with regular stirring—namely, allowing the rapid heating of aqueous samples with non-ionizing electromagnetic radiation; a lower extraction time and solvent quantities; and, in turn, a higher level of automation, allied to a superior selectivity and efficiency [22]—one of the main aims of this work was to maximize the recovery of phlorotannins from *F. vesiculosus* using the MAE technique combined with a greener solvent—namely, ethanol.

According to the preliminary single factor experiments, solvent-solid ratio was shown to have neglectable effect on the recovery of phlorotannins, contray to the ethanol concentration in the range of 0–100%, the temperature in the range of 25–150 °C, and the time in the range of 1–25 min. Concerning the effect of ethanol concentration, the maximum TPhC was obtained for an ethanol concentration of 60% (*v/v*), which is slightly higher than that settled in the optimization performed by He and colleagues [22] (55% *v/v*) for the MAE of phlorotannins from *Saccharina japonica*. Moreover, despite Magnusson et al. [12] pointed that water is a better solvent than ethanol for recovering phlorotannin with MAE, our data, combined with those of others, suggest that hydroethanolic mixtures are, in fact, more appropriate.

Afterwards, a maximum TPhC was herein obtained for the extraction temperature of 100 °C, which is higher than that established by He et al. [22] (60 °C) for *Saccharina japonica* but lower than that described by Magnusson et al. [12] (160 °C) for *Carpophyllum flexuosum*, *Carpophyllum plumosum* and *Ecklonia radiata*, using the same technique. Such differences can be explained by the thermolability of these compounds, as well as the differences in the phlorotannins profiles that probably occur between these seaweed species. In turn, the effect of time on MAE revealed that the maximum TPhC could be achieved within 3 min, which is in agreement with the work previously described by Magnusson et al. [12].

According to the BBD model, the predicted optimal conditions of extraction were set as ethanol concentration of 57% (*v/v*), temperature of 75 °C and a time of 5 min. The ethanol concentration was similar to that obtained in the preliminary single factor experiments (60% *v/v*) and to that described by He et al. [22] (55% *v/v*) for the macroalgae *Saccharina japonica*. In turn, the optimal temperature was lower than that established in the preliminary experiments (100 °C), while the extraction time was superior. These differences could be explained by the interactive effects between variables that are not considered when performing the single-factor experiments. Indeed, the results gathered from the BBD experiment allowed to conclude that the main interactions between the different variables were temperature versus time, followed by ethanol concentration versus temperature. This could be because the variables ethanol concentration and temperature revealed a quadratic effect. In the case of ethanol concentration, it is clear that the presence of water and ethanol provides a polar medium more suitable for the phlorotannin extraction than just ethanol [28]. Regarding temperature, the optimal temperature for the extraction process was set at 75 °C. To the best of our knowledge, there is no previous data focusing on the extraction of phlorotannins from *F. vesiculosus* using MAE, thus hampering the comparison of data. Naturally, the optimal conditions obtained for the MAE were quite distinct than those established by Catarino et al. [8] for the conventional solvent extraction using hydroacetonic mixtures (acetone of 70% (*v/v*), solvent-solid ratio of 70 mL/g at temperature 25 °C and time of 3 h). Nonetheless, it must be noted that the herein set conditions for MAE allowed the recovery of identical amounts of phlorotannins than conventional solvent extraction (3.16 ± 0.06 mg PGE/g DW_algae_ versus 2.94 ± 0.28 mg PGE/g DW_algae_, respectively), hence indicating that the use of MAE associated to green solvents may serve as a good alternative to the extraction of phlorotannins from *F. vesiculosus*. In fact, despite the lower concentration, the phlorotannin constituents from MAE were, in general, concordant with those from the conventional extract (as demonstrated through UHPLC-MS analysis). Regardless several non-identified compounds, it was possible to detect tetrafucol, dibenzodioxine-1,3,6,8-tetraol, pentafucol, hexafucol, heptafucol, fucofurodiphlorethol, pentafuhalol, hydroxytetrafuhalol and other distinct phlorotannin derivatives (*m/z* at 317, 623, 363, 635, 507 and 771) that were previously reported by other authors for this macroalgae species [8,29].

As phenolic compounds, phlorotannins’ most characteristic biological effect is their antioxidant activity. Indeed, several authors have proven this capacity using different approaches—namely, through anti-radical systems such as DPPH, ORAC, O_2_^•−^, and NO^•^ [30,31,32,33] and biological systems including H_2_O_2_ and *t*-BHP-induced oxidative stress [15,34] in different cell lines, and even in vivo studies on rats [30]. Overall, our findings revealed a dose-dependent activity observed for both MAE and conventional phlorotannin extracts (data not shown) with the later always showing lower IC_50_ values than the former, which is in agreement with the superior concentration of phlorotannins found in conventional rather than in MAE extract. These observations agree with previous studies supporting the evidence that the phlorotannins content of the extracts is correlated with their antioxidant capacity [32]. Nevertheless, the results gathered in this study suggest that MAE may hold antioxidant potential both through enzymatic and antiradical mechanisms, although further assays may be needed.

In addition to the antioxidant properties of phlorotannins, promising anti-diabetes effects have also been demonstrated for these compounds via the inhibition of key enzymes, prevention of the formation of advanced glycation end products, improving insulin sensitivity and others [34,35,36]. As responsible for the hydrolysis of carbohydrates into monomers of glucose, α-glucosidase constitutes an important target enzyme for the control and prevention of diabetes. Indeed, our results demonstrate that both MAE and conventional extracts of *F. vesiculosus* strongly inhibited the activity of α-glucosidase, with IC_50_ values remarkably inferior to that of acarbose, which is a pharmaceutical drug currently used to prevent the development of diabetic symptoms. Previous works showed that *F. vesiculosus* phlorotannin extracts or their purified fractions were able to strongly interfere with the activity of this enzyme [8,36,37]. Notably, consistent with the lower phlorotannin concentrations, MAE revealed lower inhibitory potential compared to the conventional extract. Nevertheless, this effect was still approximately 115 times stronger than that of acarbose, meaning that MAE combined with hydroethanolic solvent may represent a fast and safer method to obtain extracts that could help the regulation of diabetes. In any case, further elucidation of phlorotannins’ stability through the GI tract and/or bioactivity of metabolites will be required to sustain the in vivo effects.

## 4. Materials and Methods

### 4.1. Materials

Ground *F. vesiculosus* from July 2017 was purchased from Algaplus Lda (Aveiro, Portugal). Acetone, ethanol, acetonitrile HPLC grade, hydrochloric acid, and glacial acetic acid were acquired from Fisher Chemical (Pittsburgh, PA, USA). The enzymes α-glucosidase from *Saccharomyces cerevisiae* (EC No.—3.2.1.20) and xanthine oxidase from bovine milk (EC No.—1.17.3.2), together with 2,4-dimethoxybenzaldehyde (DMBA), phloroglucinol, 2,2’-azino-bis(3-ethylbenzothiazoline-6-sulphonic acid)diammonium salt (ABTS-NH_4_), nitrotetrazolium blue chloride (NBT), phenazine methosulfate (PMS), 4-nitrophenyl α-D-glucopyranoside (pNPG), allopurinol, ascorbic acid, and formic acid, were purchased from Sigma-Aldrich (St. Louis, MO, USA). Potassium persulfate, potassium di-hydrogen phosphate, potassium hydroxide, sodium di-hydrogen phosphate 1-hydrate, and gallic acid were acquired from Panreac (Barcelona, Spain). β-nicotinamide adenine dinucleotide (β-NADH), trolox, 3,5-dinitrosalicylic acid (DNS), acarbose, and 4-nitrophenol were purchased from Acros Organics (Hampton, NH, USA) and dimethylsulfoxide (DMSO) were acquired from Honeywell Riedel-de Haën (Charlotte, NC, USA), and xanthine were purchased from AlfaAesar (Ward Hill, MA, USA). All reagents were of analytical grade or of the highest available purity.

### 4.2. Methods

#### 4.2.1. Single-Factor Experiments Using Microwave-Assisted Extraction (MAE)

The method development for the extraction of seaweed phlorotannins by MAE was based on the work of He et al. [22]. The extraction process was performed by systematically varying one condition at a time—namely, the concentration of ethanol (0%, 20%, 40%, 60%, 80%, 100% (*v/v*)), the solvent-solid ratio (40, 60, 80, 100, 120, 140 and 160 (mL/g)), the extraction temperature (25, 50, 75, 100, 125 and 150 °C), and the irradiation time (1, 3, 5, 10, 15, 20 and 25 min). When one variable was not studied, it was kept constant. The constant values for irradiation time, solvent-solid ratio, temperature and microwave power were 20 min, 40 mL/g, 60 °C and 400 W, respectively, and samples were heated to the target temperature within a 2 min ramp. The extract was recovered by filtration through cotton to remove the solid residues, followed by a G4 glass filter, and was maintained at −20 °C until analysis. Experiments were performed with a focused microwave system with an Ethos MicroSYNTH Microwave Labstation (Milestone Inc.) using an 80 mL reactor at atmospheric pressure, and samples were stirred under constant agitation throughout the extraction process.

#### 4.2.2. Experimental Design for the Optimization of Phlorotannins Microwave-Assisted Extraction

An RSM based on a three-level-three-factor Box–Behnken experimental design (BBD) was employed in this study to optimize the phlorotannin extraction process considering the effects of solvent concentration (%, *v/v*, *X*_1_), temperature (°C, *X*_2_), and extraction time (min, *X*_3_). The factor levels of these three variables were coded as −1 (low), 0 (central point or middle), and +1 (high), respectively, according to the single-factor tests outlined bellow (Table 5).

A total of 15 different experiments, including three replicates at central point (Table 1), were conducted in a randomized order. Using the response surface methodology, the experimental design and analysis of variance (ANOVA) were carried out in the statistical software JMP, version 10.0.0, to generate the following second-order polynomial equation (Equation (2)) that represents the total phlorotannins content (TPhC) as a function of the coded independent variables:

(2) $$Y=\beta_{0}+\sum_{i=1}^{k}\beta_{1}X_{1}+\sum_{i=1}^{k}\beta_{ii}X_{i}^{2}+\sum_{i\neq j=1}^{k}\beta_{ij}X_{i}X_{j},$$

were *Y* is the predicted response; *β*_0_ is the constant coefficient; *β_i_*, *β_ii_*, *β_ij_* are the linear, quadratic and interactive coefficients of the model, respectively; and *X_i_* and *X_j_* are the coded independent variables.

The model adequacy was evaluated using the coefficient of determination (R^2^) and the lack-of-fit test represented at 5% level of significance, accordingly. Three-dimensional response surface plots and two-dimensional contour plots were used for the visualization of the effects of independent variables and their mutual interactions in the responses. To validate the accuracy of the models, experiments were carried out at the optimal conditions predicted for TPhC, and the obtained experimental data were compared to the values predicted by the corresponding regression model.

#### 4.2.3. Extraction of Phlorotannins under Optimal MAE and Conventional Solvent Extraction

The MAE extract was prepared following the optimal conditions determined through the response surface method. A total of 10.8 g of macroalgae (corresponding to 1.08 L hydroethanolic mixture) were used, with 60 mL in each microwave flask. The conventional solvent extraction of *F. vesiculosus* was performed according to the optimal conditions established by Catarino et al. [8]. Briefly, 11 g of dried algal powder (DW_algae_) was dispersed in 770 mL of 70% acetone solution with 1% of glacial acetic acid, and incubated for 3 h at room temperature under constant agitation. The combined mixture obtained with MAE, or by conventional solvent extraction method, was filtered through cotton to remove the solid residues and then through a G4 glass filter. Afterwards, the extract solvents were removed by rotary evaporation. The dried extracts were resuspended in DMSO and subsequently stored at −20 °C until further analysis.

#### 4.2.4. Characterization of Phlorotannins 

The TPhC was estimated according to the 2,4-dimethoxybenzaldehyde (DMBA) colorimetric method previously described [9]. Briefly, equal volumes of the stock solutions of DMBA (2%, *m*/*v*) and HCl (6%, *v/v*), both prepared in glacial acetic acid, were mixed prior to use (work solution). Afterwards, 250 μL of this solution was added to 50 μL of each extract in a 96-wells plate and the reaction was incubated in the dark, at room temperature. After 60 min, the absorbance was read at 515 nm in an automated plate reader (Biotek Instrument Inc., Winooski, VT, USA) and the phlorotannins content was determined by using a regression equation of the phloroglucinol linear calibration curve (0.06–0.1 mg/mL). The results were expressed as mg phloroglucinol equivalents per g of dried algae (mg PGE/g DW_algae_) or per g of dried extract (mg PGE/g DW_extract_).

In addition, the identification of individual phenolic compounds in the extracts was performed by UHPLC-DAD-ESI/MS analysis, after defatting with *n*-hexane, as previously described [13], and filtration through a nylon filter of 0.22 µm (Whatman™, Buckinghamshire, UK). The analysis was carried out in an Ultimate 3000 (Dionex Co., San Jose, CA, USA) apparatus consisting of an autosampler/injector, a binary pump, a column compartment, and an ultimate 3000 Diode Array Detector (Dionex Co., San Jose, CA, USA), coupled to a Thermo LTQ XL (Thermo Scientific, San Jose, CA, USA) ion trap mass spectrometer equipped with an ESI source. The LC separation was conducted with a Hypersil Gold (ThermoScientific, San Jose, CA, USA) C18 column (100 mm length; 2.1 mm i.d.; 1.9 μm particle diameter, end-capped) maintained at 30 °C and a binary solvent system composed of (A) acetonitrile and (B) 0.1% of formic acid (*v/v*). The solvent gradient started with 5–40% of solvent (A) over 14.72 min, from 40–100% over 1.91 min, remaining at 100% for 2.19 more min before returning to the initial conditions. The flow rate was 0.2 mL/min and the UV–Vis spectral data for all peaks were accumulated in the range of 200–700 nm while the chromatographic profiles were recorded at 280 nm. Control and data acquisition of MS were carried out with the Thermo Xcalibur Qual Browser data system (ThermoScientific, San Jose, CA, USA). Nitrogen of above 99% purity was used, and the gas pressure was 520 kPa (75 psi). The instrument was operated in negative mode with the ESI needle voltage set at 5.00 kV and an ESI capillary temperature of 275 °C. The full scan covered the mass range from *m*/*z* 100 to 2000. CID-MS/MS experiments were performed for precursor ions using helium as the collision gas with a collision energy of 25–35 arbitrary units. All solvents were LC-MS grade.

#### 4.2.5. Antioxidant Properties

##### ABTS^•+^ Discoloration Assay

The total antioxidant activity of both crude extracts was measured using an adaptation of the ABTS^•+^ discoloration assay based on the procedure described by Catarino et al. [13]. A stock solution of ABTS^•+^ was prepared by reacting the ABTS-NH_4_ aqueous solution (7 mM) with 2.45 mM of potassium persulfate in the dark at room temperature for 12–16 h to allow the completion of radical cation generation. This solution was then diluted with distilled water until its absorbance reached 0.700 ± 0.05 at 734 nm. Afterwards, 50 μL of each sample were mixed with 250 μL of the diluted ABTS^•+^ solution in a 96-well microplate. The mixture was then allowed to react for 20 min in the dark, at room temperature and the absorbance was then measured at 734 nm in an automated plate reader (Biotek Instrument Inc., Winooski, VT, USA). The percentage of inhibition of ABTS^•+^ was calculated using the Equation (3) described by Yen and Duh [38]:

(3) $$\%\;{\mathrm{ABTS}}^{•+}\;\mathrm{scavenging}\;=\;\frac{{\Delta A}_{c}-{\;\Delta A}_{e}}{{\Delta A}_{c}}\times 100$$

where *A*c is the absorbance of the control (without extract addition) and *A*e is the absorbance of the extract. Ascorbic acid was used as the reference compound. The concentration of the extract/standard able to inhibit 50% of ABTS^•+^ (IC_50_) was then calculated by plotting the percentage of inhibition against the plant extract concentrations.

##### Superoxide Scavenging Assay

In a 96-well plate, 75 µL of nitroblue tetrazolium (NBT) (0.2 mM), 100 µL of β-NADH (0.3 mM), 75 µL of each crude extract, and 75 µL of phenazine methosulfate (PMS) (15 µM) were mixed and incubated for 5 min at room temperature. The absorbance was then measured at 560 nm in an automated plate reader (Biotek Instrument Inc., Winooski, VT, USA). Gallic acid was used as the reference compound. The IC_50_ value for superoxide scavenging activity was determined by plotting the percentage of inhibition of superoxide radical anion generation in the presence of the crude extract and calculated using Equation (3).

#### 4.2.6. Enzymatic Assays

##### α-Glucosidase Inhibition Assay

The inhibition of α-glucosidase was measured according to the method previously described by Neto et al. [39]. In short, 50 µL of different extract concentrations (0–0.006 mg/mL in 50 mM of phosphate buffer, pH 6.8) were mixed with 50 µL of 6 mM 4-nitrophenyl-D-glucopyranoside (pNPG) dissolved in deionized water. The reaction was started with the addition of 100 µL of α-glucosidase solution and the absorbance was monitored at 405 nm every 60 s for 20 min at 37 °C. Blank readings (no enzyme) were then subtracted from each well and the inhibitory effects towards the α- glucosidase activity was calculated as follows:

(4) $$\%\;\mathrm{inhibition}\;=\;\frac{{\Delta A\mathrm{bs}}_{c}-{\;\Delta A\mathrm{bs}}_{e}}{{\Delta A\mathrm{bs}}_{c}}\times 100$$

where Δ*A*bs_c_ is the variation in the absorbance of the negative control and Δ*A*bs_e_ is the variation in the absorbance of the extract. Acarbose was used as a positive control of inhibition.

##### Xanthine Oxidase Assay

The inhibition of xanthine oxidase activity was carried out following the method described by Pereira et al. [40], with slight modifications. Briefly, in a 96-well plate 40 µL of extract (concentrations of 0–2 mg/mL) was mixed with 45 µL of sodium dihydrogen phosphate buffer (100 mM, pH 7.5) and 40 µL of enzyme (5 mU/mL). After 5 min of incubation at 25 °C, the reaction was started with the addition of 125 µL of xanthine (0.1 mM dissolved in buffer), and the absorbance at 295 nm was measured every 45 s over 10 min at 25 °C. The inhibitory effects towards xanthine oxidase activity were calculated using Equation (4). Allopurinol was used as a positive control of inhibition.

#### 4.2.7. Statistical Analysis

All the data were expressed as the mean ± standard deviation (SD) of three similar and independent experiments performed in duplicate. The JMP and Minitab software were used to construct the BBD and to analyze the results. Data from single-factor experiments and BBD were analyzed using ANOVA (*p* < 0.05), followed by Tukey’s post hoc test.

## 5. Conclusions

In this work, a single-factor experimental approach followed by a response surface methodology was carried out for the determination of the optimal conditions that maximize the extraction of phlorotannins from *F. vesiculosus* using microwave-assisted extraction combined with hydroethanolic mixtures as a solvent, as a greener approach to the conventional methods that usually make use of acetone. The optimal conditions were settled on as ethanol concentration at 57% (*v/v*), the temperature at 75 °C, and time at 5 min. When compared to the yield of extraction obtained under optimized conditions for conventional solvent extraction with hydroacetonic solvent, MAE extraction allowed the recovery of similar amounts of phlorotannins (2.94 ± 0.28 mg PGE/g DW_algae_, versus 3.16 ± 0.06 mg PGE/g DW_algae_, respectively). Likewise, the UHPLC-MS analysis revealed that both extracts presented a very similar phenolic profile, allowing the identification of 10 possible phlorotannins and seven other phlorotannin-derivatives. The two extracts were evaluated for their antioxidant properties through ABTS^•+^ and O_2_^•−^ scavenging assays and for their ability to inhibit the enzymatic activity of xanthine oxidase and α-glucosidase. In general, the conventional extract revealed better results than MAE extract, most likely due to its higher phlorotannin richness, although they both exhibited exceptional inhibitory activity against α-glucosidase, showing better results than the commercial antidiabetic pharmaceutical drug. In a wider perspective, the investigation of the applicability of seaweeds such as *F. vesiculosus* could lead to the development of nutraceuticals and pharmacological applications to treat a wide spectrum of disorders and/or diseases.

## Figures and Tables

**Table 1:** Experimental TPhC values obtained from the Box–Behnken design matrix.

Extract No.	Independent Variables			Experimental TPhC (mg PGE/g DW_algae_)
	*X*_1_	*X*_2_	*X*_3_	
1	40	125	3	1.17 ± 0.39
2	60	100	3	2.58 ± 0.36
3	80	125	3	1.61 ± 0.20
4	60	75	5	3.09 ± 0.34
5	80	100	5	1.99 ± 0.35
6	60	125	1	2.37 ± 0.25
7	80	75	3	2.16 ± 0.54
8	60	100	3	2.58 ± 0.36
9	40	100	1	2.60 ± 0.23
10	40	100	5	1.95 ± 0.37
11	60	125	5	0.85 ± 0.22
12	60	75	1	2.52 ± 0.21
13	40	75	3	2.42 ± 0.24
14	60	100	3	2.58 ± 0.36
15	80	100	1	2.35 ± 0.33

*X*_1_—ethanol concentration (%); *X*_2_—temperature (°C); *X*_3_—time (min); TPhC―total phlorotannins content. All values are expressed as mean ± SD of mg of phloroglucinol equivalents/g of dried algae (mg PGE/ g DW_algae_).

**Table 2:** Regression coefficients and results of the ANOVA analysis of the model.

Parameter	Regression Coefficient
β_0_	2.58 ***
*X*_1_	−0.004
*X*_2_	−0.52 ***
*X*_3_	−0.24 **
*X*_1_ *X*_2_	0.17 *
*X*_1_ *X*_3_	0.073
*X*_2_ *X*_3_	−0.52 ***
*X*_1_ *X*_1_	−0.36 **
*X*_2_ *X*_2_	−0.38 **
*X*_3_ *X*_3_	−0.0001
*R* ^2^	0.99
*R* ^2^_Adj_	0.96
Model *F*-value	43.77
Model *p*-value	<0.001

β_0_—constant coefficient; *X*_1_—ethanol concentration (%); *X*_2_—temperature (°C); *X*_3_—time (min). *, **, *** represent statistical significance with *p* < 0.05, 0.01, and 0.001, respectively.

**Table 3:** Phlorotannins from *F. vesiculosus* extracts obtained under optimized MAE and conventional extraction conditions.

RT (min)	[M − H]^−^	MS^2^ Main Fragments	Probable Compound	CONV	MAE
1.3	317	225, 165, 207, 125, 249, 153	Phlorotannin derivative	D	D
1.9	497	479, 331, 461, 435, 395, 165	Tetrafucol	D	D
2.5	247	203, 121, 81	Dibenzodioxine-1,3,6,8-tetraol	D	D
2.7	621	603, 455, 585, 331, 529, 559, 577	Pentafucol	D	D
4.4	745	727, 455, 579, 709, 289, 701, 683	Hexafucol	D	D
5.3	623	495, 477, 605, 577, 601, 496	Phlorotannin derivative	D	D
6.2	869	851, 579, 455, 833, 785, 703	Heptafucol	D	D
6.4	479	461, 433, 315, 389, 435, 401	Fucofurodiphlorethol	D	D
10.0	363	345, 257, 319, 138, 182	Phlorotannin derivative	D	D
11.0	637	619, 496, 593, 601, 591, 335	Pentafuhalol	D	D
11.7	497	451, 479, 437, 453, 336, 335, 461	Tetrafucol	D	D
11.8	529	485, 511, 471, 467, 493, 403, 389, 373	Hydroxytetrafuhalol	D	D
12.9	635	575, 617, 557, 335, 466, 273, 531, 229	Phlorotannin derivative	D	D
13.3	587	507, 523, 505, 383, 277, 229	Unidentified	D	D
13.5	723	679, 701, 405, 714, 497, 678, 331	Unidentified	D	D
14.2	635	575, 617, 557,335, 466	Phlorotannin derivative	D	D
14.8	587	507	Unidentified	D	D
14.8	507	277, 461, 439, 489, 479, 382, 229, 275, 231	Phlorotannin derivative	D	D
15.0	950	904	Unidentified	D	D
16.4	603	585, 559, 543, 567, 269, 523, 313	Fucofurotriphlorethol	D	D
18.5	610	225, 538, 299, 592, 226, 486	Unidentified	ND	D
19.9	771	753, 727, 761, 725, 749, 610	Phlorotannin derivative	ND	D
Total Phlorotannins (mg/g_extract_) ^(1)^				11.1 ± 1.3	9.8 ± 1.8

RT—Retention time; CONV—Conventional solvent extraction; MAE—Microwave-assisted extraction; D—detected; ND—not detected. ^(1)^ Determined by 2,4-dimethoxybenzaldehyde assay (DMBA).

**Table 4:** Antioxidant activity and inhibition of α-glucosidase of *F. vesiculosus* extracts obtained by optimized MAE and conventional methodologies.

Sample	IC_50_ (µg/mL)			
	ABTS^•+^	O_2_^•−^	Xanthine Oxidase	α-Glucosidase
MAE	95.99 ± 3.40	527.30 ± 47.78	23.07 ± 3.40	6.86 ± 0.70
Conventional	62.55 ± 1.93	457.18 ± 23.97	6.36 ± 2.20	1.73 ± 0.13
Reference compound *	5.07 ± 0.25	5.07 ± 0.77	0.05 ± 0.005	789.93 ± 41.08

MAE—Microwave-assisted extraction. IC_50_ was determined as the concentration at which ABTS^•+^ and O_2_^•−^ were inhibited by 50%. All values are expressed as mean ± SD. * Trolox was used as a reference compound for ABTS^•+^, gallic acid for O_2_^•−^, allopurinol for xanthine oxidase, and acarbose for α-glucosidase.

**Table 5:** Independent variables and their levels used in BBD.

Symbols	Independent Variables	Levels		
		−1	0	+1
*X*_1_	Solvent concentration (%, *v/v*)	40	60	100
*X*_2_	Temperature (°C)	75	100	125
*X*_3_	Time (min)	1	3	5
